# Association of Public Works Disasters with Substance Use Difficulties: Evidence from Flint, Michigan, Five Years after the Water Crisis Onset

**DOI:** 10.3390/ijerph20237090

**Published:** 2023-11-21

**Authors:** Tuviere Onookome-Okome, Angel Hsu, Dean G. Kilpatrick, Angela Moreland, Aaron Reuben

**Affiliations:** 1 Institute for the Environment, University of North Carolina Chapel Hill, Chapel Hill, NC 27517, USA; 2 Samuel Centre for Social Connectedness, Montréal, QC H3A 0G4, Canada; 3 Department of Psychiatry and Behavioral Sciences, Medical University of South Carolina, Charleston, SC 29425, USA; kilpatdg@musc.edu (D.G.K.); moreland@musc.edu (A.M.); 4 Department of Psychology & Neuroscience, Duke University, Durham, NC 27708, USA

**Keywords:** Flint water crisis, mental health, disinvestment, substance use

## Abstract

Public works environmental disasters such as the Flint water crisis typically occur in disenfranchised communities with municipal disinvestment and co-occurring risks for poor mental health (poverty, social disconnection). We evaluated the long-term interplay of the crisis and these factors with substance use difficulties five years after the crisis onset. A household probability sample of 1970 adults living in Flint during the crisis was surveyed about their crisis experiences, use of substances since the crisis, and risk/resilience factors, including prior potentially traumatic event exposure and current social support. Analyses were weighted to produce population-representative estimates. Of the survey respondents, 17.0% reported that substance use since the crisis contributed to problems with their home, work, or social lives, including 11.2% who used despite a doctor’s warnings that it would harm their health, 12.3% who used while working or going to school, and 10.7% who experienced blackouts after heavy use. A total of 61.6% of respondents reported using alcohol since the crisis, 32.4% using cannabis, and 5.2% using heroin, methamphetamine, or non-prescribed prescription opioids. Respondents who believed that exposure to contaminated water harmed their physical health were more likely to use substances to the detriment of their daily lives (RR = 1.32, 95%CI: 1.03–1.70), as were respondents with prior potentially traumatic exposure (RR = 2.99, 95%CI: 1.90–4.71), low social support (RR = 1.94, 95%CI: 1.41–2.66), and PTSD and depression (RR’s of 1.78 and 1.49, respectively, *p*-values < 0.01). Public works disasters occurring in disenfranchised communities may have complex, long-term associations with substance use difficulties.

## 1. Introduction

Public works disasters such as the Flint water crisis (FWC) typically occur in disenfranchised communities with municipal disinvestment and co-occurring risks for poor mental health (poverty, disrupted social networks) [1].

After decades of depopulation and underinvestment in infrastructure, on 25 April 2014, during a financial crisis, the city of Flint, Michigan altered its municipal water supply from Lake Huron to the Flint River [2]. This change caused water distribution pipes to corrode, allowing lead and other contaminants to enter the water delivered to homes. Although many Flint residents expressed concerns about the quality of tap water, public authorities did not issue a do-not-drink order until 15 September 2015 [2], when research by nongovernmental agencies found high blood-lead levels among Flint children [3]. In October 2015, water supplies were returned to Lake Huron, and levels of lead in tested public water returned to Environmental Protection Agency quality standards on 24 January 2017 [4]. Since that time, other public infrastructure and water contamination events have occurred or been discovered in minoritized and disinvested communities around the country, including in Jackson, Mississippi, Baltimore, Maryland [5], Newark, New Jersey [6], and New York City [7].

Public works disasters such as the Flint crisis threaten public health [8,9] and perceptions of public health [10], and therefore represent potentially traumatic events capable of precipitating psychological distress and illness [11,12]. While there is strong evidence that such events interact with pre-existing contextual factors (e.g., poverty, social disconnection) to elevate rates of diagnosable mental disorders in the community, particularly PTSD and depression [11], the interaction of these events with substance use problems remains under-investigated. As one consequence, while the Substance Abuse and Mental Health Service Administration has identified a need to incorporate the prevention and treatment of substance use problems in disaster preparedness and response programming, there remains “a scarcity of empirical information” to guide such programming, despite clear evidence that substance use is one common coping strategy employed after exposure to potentially traumatic events [13].

In Flint, survey evidence identified elevations in symptoms of PTSD [14], anxiety, and depression during the water crisis [8,15,16,17], and a high prevalence of diagnosable PTSD and major depression five years after the crisis onset [11]. In the one investigation of substance use problems, Heard–Garris et al. [18] reported an increase in substance use among youth with previous use concerns (n = 133, mean age = 26) surveyed in the second year of the crisis, with 20% of respondents endorsing the statement, “I’ve been using alcohol or other drugs to help me get through [the crisis]”.

Here we report the first Flint-wide survey of substance use difficulties following the water crisis, using mental health survey data from an area-probability household sample of adults from the Flint community surveyed five years after the crisis onset, before the COVID-19 pandemic. Descriptive statistics on the prevalence of substance use difficulties are presented alongside an analysis of the interplay of substance use difficulties with preexisting risk and protective factors and experiences related to the water crisis. Findings are intended to inform disaster response planning for public works disasters that, as with the Flint crisis, tend to occur in communities with histories of social injustice, depopulation, and disinvestment.

## 2. Materials and Methods

### 2.1. Data Collection and Sample

Data collection and sampling strategies have been previously described [11]. In brief, data were collected from August 2019 to April 2020 through web and mail-based surveys of an area-probability household sample of adults (≥age 18 years) from the Flint community, identified via address-based sampling. Letters with a study description were mailed to randomly selected households within a specified geographical area. One adult per household was randomly selected for study participation using the most recent birthday method. This adult was asked to complete a survey about their mental health and experiences related to the water crisis via mail or web. In total, 98% of survey responses were collected before 11 March 2020, when the World Health Organization declared the start of the COVID-19 pandemic, as well as before the 10 March 2020 confirmation of Michigan’s first COVID cases. Data collection thus occurred post-water crisis but pre-COVID pandemic. Survey sampling, recruitment, collection, and weighting were conducted by the national survey research firm Abt Associates. Participants gave written informed consent. The study protocol was approved by the Institutional Review Board for Human Research at the Medical University of South Carolina.

### 2.2. Survey Content

Respondents completed a self-administered, highly structured survey interview using branching-format questions. Surveys were available in English and Spanish and ascertained demographic characteristics, experiences related to the water crisis, past and current mental health symptoms, including substance use, and pre-existing mental health risk and resilience factors.

#### 2.2.1. Substance Use

*Substance use interference with daily life.* Respondents were asked six questions about substance use that interfered with home, work, or social life “since the beginning of the water crisis” in 2014, including whether they: (1) used alcohol or drugs even though a doctor suggested that they stop because of a problem with their health; (2) used substances or were high while working, going to school, or taking care of children; (3) missed or had been late for work, school, or other activities because they were drinking, using drugs, or hungover; (4) had problems getting along with other people because of their substance use; (5) experienced blackouts from use; or (6) drove a car after drinking too much or using too many drugs.

*Alcohol use.* Respondents were asked if they had consumed alcohol since the beginning of the crisis (“yes” or “no”), whether they had consumed any alcoholic beverages during the last 30 days, and on how many days (0–30) they had had four/five or more drinks within a two-hour period, and if they identified as female/male.

*Non-alcohol substance use.* Respondents were asked, “Since the Flint Water Crisis in 2014, have you ever used on four or more occasions”: marijuana or pot, heroin, methamphetamine, or non-medically prescribed codeine, Darvon, Percodan, Demerol, morphine, or OxyContin.

#### 2.2.2. Correlates of Substance Use

*Experiences related to the water crisis.* Respondents were asked whether they lived in homes directly affected by tap water quality, experienced physical health problems related to exposure to contaminated water, or knew someone who had, or experienced emotional concerns related to worries about tap water quality. They were also asked about their confidence in information about water safety provided by government officials during and after the crisis.

*Pre-existing risk factors unrelated to the water crisis*. Past exposure to potentially traumatic events and low levels of social support were considered potential risk factors for substance use that were unrelated to the water crisis. Potentially traumatic event exposures were assessed in the PTSD module described below. Low levels of social support were measured using a modified five-item version of the Medical Outcomes Study module that assesses social support over the past 6 months. Respondents were asked about how often they had an individual who would “help you if you were confined to bed,“ “give good advice about a crisis,” “get together with for relaxation,” “confide in or talk with about your problems,” and “love you and make you feel wanted.” Responses ranged from “none of the time” (score: 1) to “all of the time” (score: 4) (total scale range: 5–20; low social support defined as a score of ≤15).

*Diagnosable mental disorder (PTSD and depression).* Major depression was assessed via the APA Diagnostic and Statistical Manual, Fifth Edition (DSM-5) criteria for major depressive episodes using a modified version of the National Women’s Study Depression Module previously used in NIH-funded epidemiological surveys [11], and assessed all nine symptoms of a major depression episode. Criteria were met if respondents reported a depressed mood most of the day, nearly every day, or markedly diminished pleasure or interest in all or most activities most of the day, with a total of greater than or equal to 5 depression symptoms (e.g., changes to mood, sleep, appetite). PTSD was measured via the National Stressful Events Survey (NSES) PTSD Module developed in conjunction with the APA DSM-5 PTSD Workgroup [11]. This NSES module measures exposure to any of the 11 DSM-5 potentially traumatic events, including life-threatening accidents, medical conditions, or physical or sexual assault, and all 20 DSM-5 PTSD symptoms as well as whether symptoms have resulted in significant distress or impairment. Both modules determine whether diagnostic criteria were met in the past month, past year, or any other time during their life.

### 2.3. Statistical Analyses

The study analyses followed two steps. First, the prevalence of substance use difficulties in the Flint community in the 5 years since the water crisis onset was determined, defined as substance use that interferes with daily life at home, work, or school. Descriptive statistics on the type of substances consumed were also generated. Second, we investigated potential correlates of substance use via Poisson regression models with robust error-variance [19] regressing study outcomes onto four categories of correlates: (1) sociodemographic correlates (income, race, and sex) (age was not assessed); (2) water crisis-related psychological risk factors (concerns about contaminated-water exposure-related health problems and low confidence in the information provided by government officials); (3) psychological risk factors unrelated to the crisis (past trauma exposure and low levels of social support); and (4) diagnosable mental disorder (PTSD and depression). Study analyses were designed to be primarily descriptive and aimed to provide evidence on the covariance of substance use difficulties among Flint residents with the four categories of correlates. Associations of the study outcomes with the psychological risk factors and disorders (correlates 3, 4, and 5) were adjusted for the sociodemographic factors of income, race, and sex in order to identify covariance among these factors over and above the well-described potential contributions of sociodemographics [20]. The resulting statistical effect estimates are not assumed to be causal per se. Because alcohol consumption was so prevalent (61.1% of respondents), correlate tests investigated a subset of more intense use, defined as individuals who endorsed binge drinking at least once over the past month (21.9% of respondents).

Data analyses were weighted to adjust for potential nonresponse bias due to nonparticipation and to produce population-representative estimates by first weighting to adjust for household size and likelihood of household nonresponse, and then using iterative proportional fitting to align the characteristics of the sample to match the full Flint population profile on sociodemographic characteristics of race/ethnicity, sex, marital status, education levels, and household size based on the U.S. Census Bureau 2018 American Community Survey’s 1-year estimates for Flint, Michigan. This report follows STROBE Guidelines for the reporting of cross-sectional studies.

## 3. Results

In total, 10,000 addresses were sampled and mailed recruitment letters following area probability sampling. Of these, 6693 letters received no response. Another 1112 letters were identified as undeliverable or delivered to vacant addresses. The remaining 2195 households accessed the survey, reviewed the consent materials, and completed the eligibility screening questions. Of these, 1970 (54.5% female) completed the survey. Further details on refusals, screen-outs, and response rates are described in Reuben et al. [11]. After considering estimates of household ineligibility to respond (e.g., addresses with residents only under the age of 18 years, vacant addresses, etc.), the overall estimated response rate was 28.4%; however, the response rate among those who reviewed the consent materials was 89.7%. The final weighted sample was demographically representative of the adult Flint population (Appendix A Table A1). The majority of respondents self-identified as Black or African American (53.5%) and non-Hispanic (97.4%), while 42.5% self-identified as White. A majority (56.8%) of respondents reported earning <$25,000 a year.

### 3.1. Perceived Harm from the Water Crisis

As previously reported [11], 86.8% of respondents lived in homes during the crisis that were directly affected by problems with tap water quality, and nearly all (97.7%) altered their behavior to limit their exposure to contaminated water. This included avoiding cooking (91.9%), cleaning with (47.0%), or drinking (78.0%) tap water. Despite efforts to avoid contaminated water exposure, most respondents felt that their own health (75.3%) or their family’s health (73.8%) was affected by exposure. The majority of respondents (80.1%) were concerned about potential long-term health consequences for themselves or their family members due to potential exposure to contaminated water.

### 3.2. Substance Use

**Figure 1:**
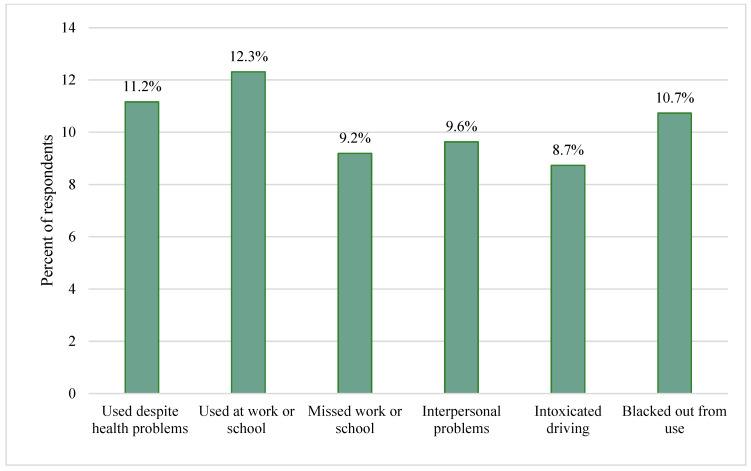
Prevalence of substance use interference with daily life among Flint residents in the 5 years following the water crisis onset.

*Use interference with daily life.* At the time of the survey five years after the water crisis onset (2019 to 2020), substance use difficulties were prevalent among Flint residents (Table 1). One in six respondents (17.0%) reported that substance use since the water crisis contributed to problems with their home, work, or social life (Figure 1), including 11.2% who used substances despite a doctor’s warnings that it would harm their health, 12.3% who used while working, going to school, or taking care of children, and 10.7% who experienced blackouts after heavy drinking or substance use.

*Alcohol consumption.* A total of 61.6% of respondents reported having consumed alcohol since the start of the water crisis, 71.4% of whom reported alcohol consumption in the past 30 days. Of those who drank in the past month, 49.8% endorsed binge drinking at least once (21.9% of the full sample) (Table 1). Of those who endorsed binge drinking, most (30.9%) did so only one to four times in the past month (approximately once per week). However, a significant portion (18.9%) did so five or more times (approximately twice per week), and a full 10% did so nine times or more.

*Marijuana and unsanctioned substance use.* One in three respondents (32.4%) reported having used marijuana on at least four occasions since the water crisis onset. One in twenty (5.2%) reported having used heroin, methamphetamine, or any prescription opioids for non-medical purposes on at least four occasions since the water crisis onset.

*Correlates of use.* Sociodemographic factors were significantly associated with substance use among Flint residents in the five years following the water crisis onset. Men were more likely than women to binge drink (RR = 1.27, 95%CI: 1.01–1.58), use marijuana (RR = 1.39, 95%CI: 1.19–1.61), and use substances to the detriment of their daily lives (RR = 1.71, 95%CI: 1.36, 2.14) (Table 1), although they were not more likely to use unsanctioned substances (RR = 0.95, 95%CI: 0.61–1.49). Low-income respondents (those reporting household income of less than $25,000) were much more likely than higher-income groups to use marijuana (RR = 1.56, 95%CI: 1.32–1.85) and unsanctioned substances (RR = 2.05, 95%CI: 1.23–3.42) and to use substances to the detriment of their daily lives (RR = 1.33, 95%CI: 1.04–1.71). (Respondent age was not assessed.)

Table 2 reports the association of substance use with the three pre-defined correlates of substance use that were non-sociodemographic, including experiences related to the water crisis, pre-existing psychological risk factors, and diagnosable mental disorders (PTSD and depression).

The analysis of the non-sociodemographic correlates identified that water crisis-related experiences were significantly associated with substance use in the five years following the water crisis onset. Respondents who believed that their health was harmed by exposure to contaminated water were more likely to use marijuana (RR = 1.31, 95%CI: 1.12–1.55) and to use substances to the detriment of their daily lives (RR = 1.32, 95%CI: 1.03–1.70). Respondents who reported low confidence in government-official information provided about the water crisis were more likely to use marijuana (RR = 1.33, 95%CI: 1.11–1.59). Neither group was more likely to binge drink or use unsanctioned substances.

Pre-existing psychological risk factors were also significantly associated with substance use (Table 2). Respondents with past exposures to potentially traumatic events in addition to the water crisis, such as natural disasters, combat, or life-threatening medical conditions, had a 63% higher risk for binge drinking (RR = 1.63, 95%CI: 1.18–2.27), 108% higher risk for marijuana use (RR = 2.08, 95%CI: 1.59–2.71), 291% higher risk for unsanctioned substance use (RR = 3.91, 95%CI: 1.55–9.87), and 199% higher risk for substance use to the detriment of their daily lives (RR = 2.99, 95%CI: 1.90–4.71). Separating potentially traumatic exposures into those specifically related to sexual or physical assault versus all others indicated that, as has been previously shown [11], physical/sexual assault are particularly potent risk factors. Those who were exposed to physical/sexual assault had a nearly five-fold higher risk for unsanctioned substance use (RR = 4.88, 95% CI: 1.88–12.67) and a four-fold higher risk for substance use to the detriment of their daily lives (RR = 4.11, 95%CI: 2.60–6.50). Respondents reporting low social support, meaning few to no people to confide in, relax with, or ask for advice, were also at greater risk for substance use in all categories of use (RR’s from 1.24 to 1.94), although associations were non-statistically significant for binge drinking (RR = 1.29, 95%CI: 0.97–1.71).

Finally, concurrent diagnosable mental disorder (PTSD and depression) was also significantly associated with substance use (Table 2). Respondents who met DSM-5 criteria for PTSD or depression based on their survey responses were significantly more likely to binge drink, use marijuana, use unsanctioned substances, and use substances to the detriment of their daily lives (RR’s from 1.49 to 3.34, *p*-values < 0.01).

## 4. Discussion

This cross-sectional population-representative survey of adult Flint residents taken 5 years after the start of the Flint water crisis yielded two principal findings. First, substance use difficulties were prevalent in the Flint community in the years following the water crisis. One in six survey respondents endorsed substance use that interfered with their home, work, school, or social lives. Most respondents (61.6%) reported using alcohol since the crisis and many (32.4%) reported using cannabis. A significant minority (5.2%) reported using heroin, methamphetamine, or non-prescribed prescription opioids. In some cases, these prevalence rates exceed benchmarks from other similar populations. For example, rates of marijuana use in Flint (32.4%) are higher than those reported for past-year use in Michigan overall during the study period (23.6%), although they are similar to those reported from other major urban areas (e.g., Washington D.C.’s rate of 30.8%) [22]. Likewise, past-month binge drinking rates in Flint (21.9%) are higher than those reported for Michigan overall (16.1%) and the wider US (15.4%), although they are, again, similar to those reported for Washington D.C. (22.5%) [23].

Second, patterns of substance use in Flint reflected known or suspected risk trends related to sociodemographics, water crisis-related experiences, pre-existing psychological risk factors, and diagnosable mental disorders. In Flint in the years following the water crisis, men and individuals with a low income had a greater prevalence of use across most, though not all, categories of use. While individuals’ experiences during the crisis (their concerns about long-term health consequences of exposure to contaminated water and their confidence in the information provided by government officials) were largely unrelated to substance use, individuals with greater crisis-related health concerns were more likely to endorse use that interfered with their daily lives.

As in past studies [11], pre-existing risk factors for substance use difficulties (past exposure to potentially traumatic events and low social support) were found to be potent risk factors in this setting. While past exposure to potentially traumatic events, particularly sexual and physical assault/abuse, was highly prevalent among Flint respondents (75.1% reported past exposure to at least one potentially traumatic event, of whom 52.1% reported sexual or physical assault/abuse), these rates are not abnormal. In fact, our Flint estimates closely match those of the wider U.S. (89.7% of U.S. respondents on a similar survey reported past exposure to at least one potentially traumatic event, of whom 53.1% reported sexual or physical assault/abuse). The very high prevalence of low social support in the Flint community (68.5%) does, however, reflect a deviation from past findings from similar surveys among other communities assessed in the U.S. after disasters, including the 9/11 terrorist attacks (38.2% of respondents reported low social support) [24] and hurricanes in Florida (36.4% of respondents reported low social support) [25]. These specific findings warrant further research attention, but may be related to the known tendency for communities burdened with historic disinvestment and/or inequitable housing practices, such as redlining and blockbusting, to have low levels of social cohesion [26]. It may also reflect changes in the community related to the water crisis itself, which appears to have lowered perceived trust in government officials [27] while, in contrast to the current findings, strengthening social networks [28].

Finally, psychiatric symptoms numerous and severe enough to warrant diagnoses of PTSD and depression, which were highly prevalent in the community (rates of 24.4% and 22.1%, respectively), were also significant correlates of substance use in Flint. This likely reflects the complex interplay of these symptoms and behaviors that tends to make them highly comorbid. (For example, for individuals experiencing PTSD, the prevalence of substance use difficulties ranges from 19% to 52% [29].) The overlap among these conditions could be explained by a common cause (e.g., exposure to a traumatic event), shared genetic risk, or a causal pathway whereby one condition elevates risk for another (e.g., problematic substance use may heighten the risk of experiencing multiple traumatic events and thus increase the risk of PTSD over time) [29].

Within the wider literature, our findings may be best contextualized by the social vulnerability framework depiction of difficulties in communities following disasters. This framework views disasters not as random events but rather as “the result of predictable hazards that interact with preexisting and long-established social relations and power dynamics that stratify resources” [13]. Hence, while flash floods and hurricanes may be acts of weather, the resulting physical, economic, and psychiatric harm they cause results from resource disparities that follow sociodemographic differences in race/ethnicity, gender, age, and class. Substance use difficulties have been previously found to rise after disasters, as individuals are believed to use substances to cope with the stress and psychological sequela of the event. This tendency has been reported in studies of the 9/11 terrorist attacks, earthquakes in Japan, hurricane Sandy in New York, and tsunamis in Southeast Asia [13]. While elevations in substance use appear to subside over a period of months for most users, a small subset appears to maintain new, higher levels of use post-disaster, on the order of years [13,30,31,32]. Echoing our findings, these users tend to be more likely to meet criteria for PTSD [32]. While our study cannot differentiate between new or expanded substance users, our findings reinforce the perspective that socially vulnerable communities are likely to experience non-transient elevations in substance use difficulties after disasters.

This study has limitations. First, our assessment of substance use was limited. We did not assess all 11 criteria symptoms of DSM-5 substance use disorder (SUD) and thus could not determine the prevalence of diagnosable SUD in the Flint community. Second, the overall response rate was only 28.4%, although 89.7% of those who read the consent agreement agreed to participate and completed the survey. While responses were weighted to match Flint’s demographic characteristics, our estimates may be low if those with substance use difficulties were less likely to respond to the survey, or high if those with difficulties were more likely to participate. Unfortunately, this study’s response rate is consistent with most large-scale community surveys conducted in the past several years, which have shown consistent declines in response rates over the past three decades [33]. To deal with this common time-of-survey effect, response weighting is believed to effectively address the issue of potential nonresponse bias in outcome distributions due to lower response rates [34,35]. Finally, data were cross-sectional and could not establish temporal or causal associations among study variables; we did not ask respondents about their substance use before the crisis.

## 5. Conclusions

Members of the Flint community have shown remarkable resilience in the years following the water crisis. Nevertheless, community members experienced high rates of substance use interference with daily life in the years following the crisis onset. Such use overlapped with pre-existing risk factors that tend to co-occur in disinvested communities, including high rates of past exposure to potentially traumatic events, low social support, and diagnosable mental disorders, as well as experiences related to the water crisis itself. Public works environmental disasters occurring in disenfranchised communities may have complex, long-term associations with substance use difficulties.

## Figures and Tables

**Table 1:** Weighted prevalence and sociodemographic correlates of substance use in Flint in the 5 years following the water crisis.

	Substance Use Interference with Daily Life Since the Crisis Onset ^a^	Past Month Binge Drinking ^b^	Marijuana Consumption Since the Crisis Onset ^c^	Methamphetamine, Heroin, or Non- Prescribed Opioid Use Since the Crisis Onset ^d^
**Prevalence, No. (%)**	335 (17.0)	432 (21.9)	638 (32.4)	102 (5.2)
**Sociodemographic correlates**	**RR (95% CI)**			
Race				
Black	0.98 (0.77–1.25)	0.96 (0.76–1.21)	0.86 (0.74–1.01)	1.20 (0.76–1.90)
>1 Race	1.32 (0.81–2.15)	1.24 (0.68–2.29)	0.91 (0.62–1.34)	1.76 (0.71–4.33)
Other	1.37 (0.72–2.60)	1.47 (0.83–2.61)	0.87 (0.46–1.64)	1.29 (0.53–3.15)
White	1 [Reference]	1 [Reference]	1 [Reference]	1 [Reference]
Sex				
Female	1 [Reference]	1 [Reference]	1 [Reference]	1 [Reference]
Male	**1.71 (1.36–2.14)**	**1.27 (1.01–1.58)**	**1.39 (1.19–1.61)**	0.95 (0.61–1.49)
Income, per year. $				
≥$25,000	1 [Reference]	1 [Reference]	1 [Reference]	1 [Reference]
<$25,000	**1.33(1.04–1.71)**	1.17 (0.94–1.48)	**1.56 (1.32–1.85)**	**2.05 (1.23–3.42)**

*Notes.* Risk ratios (RR) reflect statistical effect estimates from Poisson regression models regressing the substance use outcomes onto each sociodemographic correlate individually. CI = confidence intervals. Statistically significant effect estimates are highlighted in bold text. ^a^ Respondents who responded yes to at least one of six questions about substance use interference with daily life since the beginning of the water crisis. ^b^ Respondents who endorsed drinking four/five or more drinks during a two-hour period in the last 30 days if they identified as female/male. ^c^ Respondents who endorsed using marijuana on at least four or more occasions since the beginning of the water crisis. ^d^ Respondents who endorsed using heroin, methamphetamine, or opiates for non-medical purposes on at least four or more occasions since the beginning of the water crisis.

**Table 2:** Correlates of substance use in Flint in the 5 years following the water crisis that are related to the water crisis, reflect pre-existing psychological risk factors, or are diagnosable mental disorders.

	Substance Use Interference with Daily Life Since the Crisis Onset	Past Month Binge Drinking	Marijuana Consumption Since the Crisis Onset	Methamphetamine, Heroin, or Non- Prescribed Opioid Use Since the Crisis Onset
	**RR (95% CI)**			
**Water crisis-related correlates**				
Believe that health was harmed by exposures ^a^	**1.32 (1.03–1.70)**	1.06 (0.84–1.33)	**1.31 (1.12–1.55)**	0.97 (0.62–1.53)
Have low confidence in official information ^b^	0.99 (0.77–1.28)	1.16 (0.90–1.47)	**1.33 (1.11–1.59)**	0.94 (0.58–1.53)
**Pre-existing psychological risk factor correlates**				
Past exposures to any potentially traumatic events ^c^	**2.99 (1.90–4.71)**	**1.63 (1.18–2.27)**	**2.08 (1.59–2.71)**	**3.91 (1.55–9.87)**
Exposure to physical/sexual assault/abuse ^c^	**4.11 (2.60–6.50)**	**1.90 (1.34–2.69)**	**2.47 (1.88–3.24))**	**4.88 (1.88–12.67)**
Exposure to a non-assault traumatic event ^c^	**1.71 (1.02–2.84)**	1.35 (0.93–1.94)	**1.59 (1.18–2.14)**	**2.73 (1.03–7.25)**
Low social support ^d^	**1.94 (1.41–2.66)**	1.29 (0.97–1.71)	**1.24 (1.02–1.50)**	**1.81 (1.02–3.22)**
**Diagnosable mental disorder correlates**				
PTSD ^e^	**1.78 (1.42–2.23)**	**1.92 (1.66–2.23)**	**2.41 (2. 14–6.15)**	**3.34 (2.64–4.27)**
Depression ^e^	**1.49 (1.18–1.87)**	**1.60 (1.38–1.86)**	**2.69 (1.72–4.21)**	**2.72 (2.17–3.42)**

*Notes.* Risk ratios (RR) reflect statistical effect estimates from Poisson regression models regressing the substance use outcomes onto each correlate individually. All associations are adjusted for the sociodemographic characteristics of income, race/ethnicity, and sex (Table 1). CI = confidence intervals. Statistically significant effect estimates are highlighted in bold text. ^a^ Respondents with health concerns that they or their family members were moderately or greatly harmed by exposure to contaminated water (50.9%). ^b^ Respondents who reported low confidence in information on water safety provided by government officials during and after the water crisis (65.7%). ^c^ Respondents with past exposure to a potentially traumatic event (75.1%) compared to those without such exposure. Of those with previous exposure, 52.1% were exposed to sexual or physical assault/abuse, and 47.9% were exposed only to other forms of potentially traumatic events (e.g., combat, motor vehicle accidents). These prevalence rates closely match those of the wider U.S. [21]. ^d^ Respondents scoring less than or equal to 15 on a 5- to 20-point scale measuring social support (68.5%). ^e^ Respondents meeting DSM-5 criteria for PTSD (24.4%) and major depression (22.1%).

## Data Availability

Data reported in this article are available to qualified researchers upon reasonable request to the authors.

## Appendix A

**Table A1:** Weighted sociodemographic characteristics of the study participants.

Variable	No.	%
**Sex**	**1946**	
Female	1061	54.5%
Male	885	45.5%
**Education**	**1965**	
Less than high school diploma	295	15.0%
High school diploma/GED	760	38.7%
Some college/associate’s degree	654	33.3%
4-year college graduate	163	8.3%
Post-graduate training	92	4.7%
**Race**	**1951**	
Asian	2	0.1%
Black or African American	1043	53.5%
Native American	5	0.3%
More than one	41	2.1%
Other	31	1.6%
White	829	42.5%
**Hispanic ethnicity**	**1946**	
Hispanic	1895	97.4%
Non-Hispanic	51	2.6%
**Marital status**	**1950**	
Married	552	28.3%
Divorced/separated	398	20.4%
Widowed	136	7.0%
Never married	864	44.3%
**Household size**	**1899**	
1-person household	460	24.2%
2-person household	682	35.9%
3-person household	347	18.3%
4+person household	410	21.6%
**Annual income**	**1836**	
Less than $25,000	1043	56.8%
$25,000–$49,999	499	27.2%
$50,000–$74,999	178	9.7%
$75,000–$99,999	55	3.0%
$100,00 or more	61	3.3%

*Note*. Demographic characteristics are weighted to match US Census Bureau 2018 American Community Survey one-year estimates for demographics of adults in Flint, Michigan.
